# Nuclear localisation of Aurora-A: its regulation and significance for Aurora-A functions in cancer

**DOI:** 10.1038/s41388-021-01766-w

**Published:** 2021-05-13

**Authors:** Francesco Davide Naso, Dalila Boi, Camilla Ascanelli, Georgiana Pamfil, Catherine Lindon, Alessandro Paiardini, Giulia Guarguaglini

**Affiliations:** 1 Institute of Molecular Biology and Pathology, National Research Council of Italy, c/o Sapienza University of Rome, Rome, Italy; 2 Department of Biochemical Sciences, Sapienza University of Rome, Rome, Italy; 3 Department of Pharmacology, University of Cambridge, Cambridge, UK

**Keywords:** Cancer stem cells, Drug development, Oncogenes, Cell division

## Abstract

The Aurora-A kinase regulates cell division, by controlling centrosome biology and spindle assembly. Cancer cells often display elevated levels of the kinase, due to amplification of the gene locus, increased transcription or post-translational modifications. Several inhibitors of Aurora-A activity have been developed as anti-cancer agents and are under evaluation in clinical trials. Although the well-known mitotic roles of Aurora-A point at chromosomal instability, a hallmark of cancer, as a major link between Aurora-A overexpression and disease, recent evidence highlights the existence of non-mitotic functions of potential relevance. Here we focus on a nuclear-localised fraction of Aurora-A with oncogenic roles. Interestingly, this pool would identify not only non-mitotic, but also kinase-independent functions of the kinase. We review existing data in the literature and databases, examining potential links between Aurora-A stabilisation and localisation, and discuss them in the perspective of a more effective targeting of Aurora-A in cancer therapy.

## Introduction

Aurora kinases are a family of serine/threonine kinases essential for mitotic execution in all eukaryotes. The centrosome localised vertebrate member of the family, Aurora-A, was originally named STK15/BTAK (Breast Tumour Amplified Kinase) due to its overexpression in breast cancer [1]. Aurora-A is involved in the centrosome maturation process and contributes to the activation of the PLK1 kinase, thus promoting the transition from G2 to mitosis [2, 3]. Aurora-A then exerts its control of mitotic progression by phosphorylating several targets. Upon autophosphorylation on Thr288, along with binding to activating partners among which TPX2 plays a major role, Aurora-A acquires a fully active conformation and regulates the assembly of the mitotic spindle from both centrosomes and chromosomes [3, 4]. Mitotic roles of Aurora-A are widely studied and have been extensively reviewed elsewhere (see for example [3, 5]), while interphase functions in non-transformed and cancer cells are emerging [6].

Aurora-A undergoes cell cycle regulation, with increased levels in late S and G2 phases, and a peak in mitosis, followed by proteasome-dependent degradation at mitotic exit; nonetheless, recent evidence points out the existence of an interphase pool of Aurora-A in specialised, non-transformed cells, exerting physiological non-mitotic functions at the G0/G1 phases of the cell cycle. It is involved in primary cilium disassembly in ciliated cells, neurite outgrowth in post-mitotic neurons and in the formation of the DNA pre-replication complex (recently reviewed by [6]). Aurora-A also regulates mitochondrial morphology and dynamics throughout the cell cycle [7, 8], through a phosphorylation cascade that includes RALA/RALBP1 and cyclin B/CDK1 and that culminates in the phosphorylation of DRP1, a protein acting in mitochondrial fragmentation.

Aurora-A increased levels have been reported in various types of cancer such as neuroblastoma, lymphoma, breast, colorectal, ovarian and prostate cancers [9, 10]. Indeed, since its discovery, it was proposed as an oncogene based on the observation that its overexpression was sufficient to induce NIH/3T3 murine cell transformation [11], although subsequent studies have highlighted the relevance of the cellular background for Aurora-A’s transforming potential (reviewed by [12]). Although amplification of the chromosomal region 20q13, where *AURKA* gene is located, frequently occurs in cancer cells [12–14], transcriptional and/or post-translational alterations can also represent possible routes to increased Aurora-A levels [15, 16]. High Aurora-A levels were shown to correlate with highly proliferative cancers, with epithelial-mesenchymal transition [15, 17], with drug-resistance [18–20] and tumour metastasis formation [15, 21]. The relevance of Aurora-A in cancer has driven efforts aimed at exploiting the kinase as therapeutic target: different kinase activity inhibitors (ATP-competitive) are currently under investigation in clinical trials, although poor efficacy has been shown despite promising preclinical data [19, 22, 23].

Interestingly, when Aurora-A is deregulated, it acquires cancer-related roles that do not correspond to physiological Aurora-A functions in normal conditions. Here we will discuss recent evidence of non-mitotic nuclear and kinase-independent roles of Aurora-A in cancer progression and cancer cell stemness maintenance. We will analyse which regulatory layers may underlie these interphase roles of Aurora-A and how they can be affected in cancer. Finally, we will explore the intriguing possibility that therapeutic approaches based on Aurora-A inhibition are not targeting the relevant pool of the kinase in these contexts, opening the way for improved strategies that would take into account its non-mitotic oncogenic functions.

## Aurora-A nuclear localisation is required for oncogene-mediated cell transformation and self-renewal of cancer stem cells

**Fig. 1:**
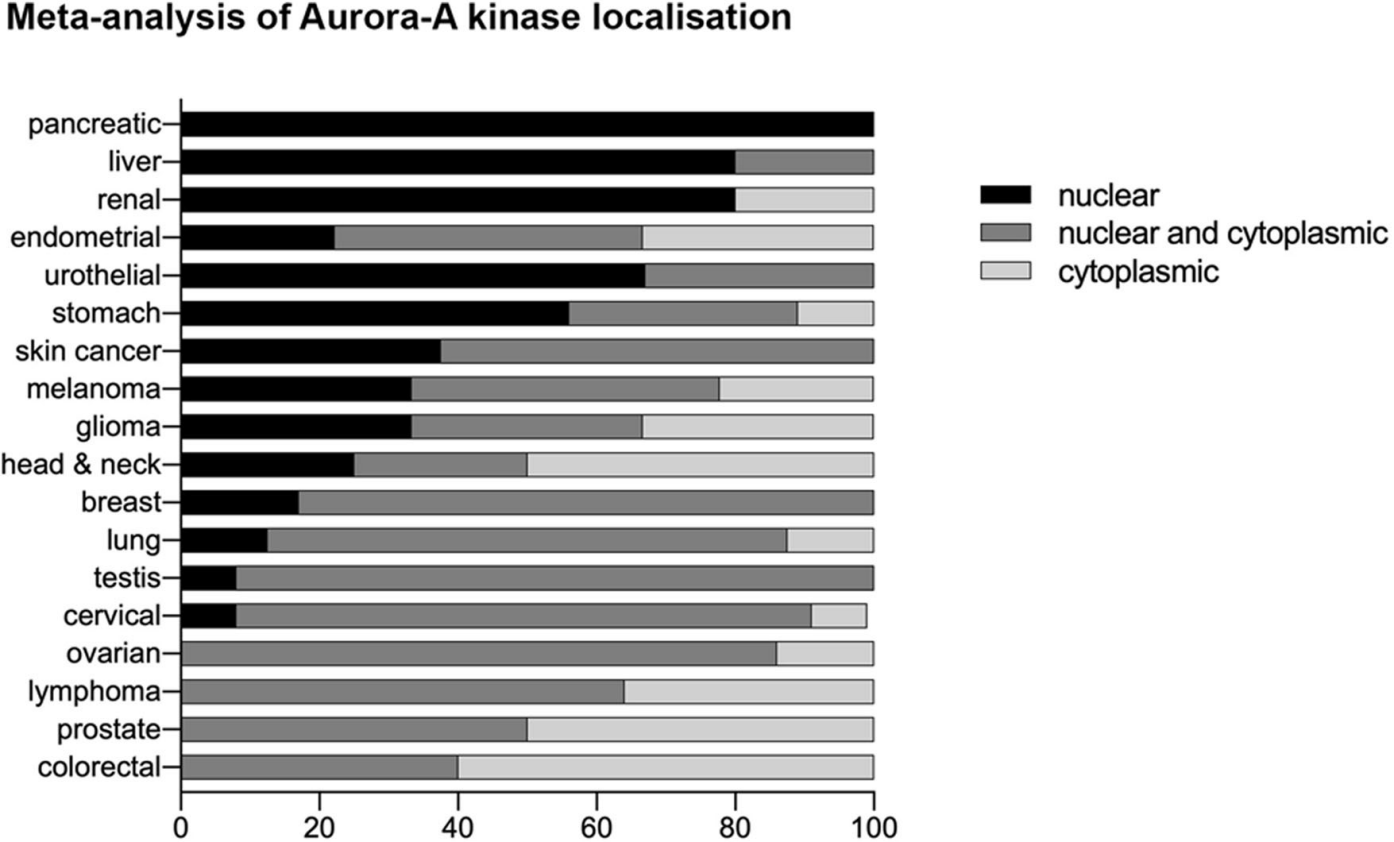
Meta-analysis of Aurora-A localisation. Locations scored as ‘nuclear’, ‘cytoplasmic/membranous’ or ‘cytoplasmic/membranous and nuclear’ in the database (here we have removed the designation ‘membranous’ as misleading) are expressed as percentage of total Aurora-A-positive samples for each tumour type (*n* ≥ 4).

Evidence of Aurora-A nuclear localisation in cancer exists in cell lines from solid and haematological tumours [24–28] and interestingly, Aurora-A appears to be overexpressed in all of these conditions. Aurora-A localisation in six head and neck cancer cell lines was assessed by western blot on the nuclear fraction and by immunofluorescence and was found inside the nucleus, which differs from its localisation in non-transformed keratinocytes, where it is only centrosomal [27]. Nuclear Aurora-A was also observed in breast cancer, specifically in five patient-derived primary cultures from breast cancer tissue and four breast cancer cell lines (MDA-MB-231, Sk-br-3, SUM149 and BT549) [28]. Within the pathology database of the Human Protein Atlas (https://www.proteinatlas.org/humanproteome/pathology) we found widespread differences in apparent distribution of Aurora-A between nuclear and cytoplasmic compartments in the histological samples recorded, with certain cancer types scoring more highly for nuclear localisation—notably pancreatic, renal and liver—than others (Fig. 1). Interestingly, we found that in the accompanying study matching cancer transcriptomics to patient survival data [29], three of the four cancer subtypes in which Aurora-A overexpression was found to be prognostic were those showing the highest tendency for exclusive nuclear localisation of Aurora-A. Interestingly, nuclear Aurora-A has been proposed as a prognostic marker for poor survival in breast cancer [24].

Specific experimental approaches where localisation can be modulated were undertaken to directly address whether this particular localisation contributes to Aurora-A oncogenic ability. Tatsuka et al. [27] report that a fusion protein Aurora-A-NES (Nuclear Export Signal) is actively exported outside the nucleus. In BALB/c 3T3 A31-1-1 murine fibroblasts, Aurora-A wild type, but not Aurora-A-NES, was able to increase the frequency of oncogenic H-Ras^G12V^-induced transformation, indicating the relevance of Aurora-A nuclear localisation [27]. Interestingly, K/H-Ras^G12V^ mutants were able to yield high Aurora-A levels in lung, ovarian and pancreatic cancer cells [30–32], as well as in primary mouse embryonic fibroblasts, where this intriguingly corresponded to a significantly increased Aurora-A nuclear/cytoplasmic ratio when compared to non-transformed murine cells [28]. Together these observations suggest a positive regulatory loop between K/H-Ras and Aurora-A, promoting cell transformation.

A more complex system to address the oncogenic role of nuclear Aurora-A was used in breast cancer cell lines, where Aurora-A depletion by RNA-interference (RNAi) results in reduction of mammosphere formation and breast cancer stem cells (BCSCs), identified as the CD24^low^/CD44^high^ cell population [28]. In order to distinguish the contribution of nuclear Aurora-A in the regulation of the BCSC phenotype, a fusion protein constituted by Aurora-A, the hormone-binding domain of oestrogen receptor (ER) and a NES sequence (Aurora-A-ER) was generated, which was reported to retain kinase activity (as assessed by p-p53 and p-H3 signals [28]). In this study, Aurora-A-ER localises in the cytoplasm, but, after the administration of 4-hydroxytamoxifen (OHT), the fusion protein re-localises inside the nucleus: this strategy allows to control the accumulation of nuclear Aurora-A. Importantly, only the nuclear translocation of Aurora-A-ER, upon 4-OHT administration, was able to restore the CD24^low^/CD44^high^ population and mammosphere formation in Aurora-A-depleted MDA-MB-231, SUM149 and BT549 Triple Negative Breast Cancer (TNBC) cells, indicating that Aurora-A nuclear localisation is required for breast cancer proliferation and staminal potential [28]. Interestingly, Aurora-A kinase inhibitors were not effective in suppressing the CD24^low^/CD44^high^ population in MDA-MB-231 cells silenced for endogenous Aurora-A and expressing exogenous Aurora-A-NLS (Nuclear Localisation Signal) [28], suggesting that nuclear Aurora-A exerts kinase-independent functions.

Taken together, these data highlight the existence of a nuclear pool of Aurora-A in cancer cells and lay the foundations for the comprehension of new mechanisms of action, possibly kinase independent, through which Aurora-A overexpression contributes to the transformed phenotype.

## Aurora-A as transcriptional regulator and target of Myc and FOXM1 in breast cancer

With the intent of clarifying the ‘non-canonical’ nuclear interphase roles of Aurora-A in cancer stem cell self-renewal, the intriguing possibility that the kinase could work as a transcriptional regulator in breast cancer was investigated [28, 33]. Expression of a chimeric version of Aurora-A fused to the GAL4-DNA-binding domain was able to yield the expression of a luciferase reporter starting from a minimal promoter displaying GAL4-DNA-binding sites, highlighting Aurora-A’s transactivation activity [28]. Authors identified a nine amino acid sequence (9aa TAD), a domain that is already known to elicit transactivation activity [34], in the region 238-246 of Aurora-A [28] within the kinase domain, and showed that it is required for Aurora-A transactivating functions.

In the search for target genes regulated by nuclear Aurora-A, it was noted that nuclear overexpressed Aurora-A is associated with upregulation of Myc and FOXM1 proteins and mRNA levels in the BSCS CD24^low^/CD44^high^ population, as well as of the stem cell markers SOX2 and NANOG [28, 33]. The well-known oncogenes Myc and FOXM1 are transcription factors normally involved in cellular growth: in particular the former is a ‘master regulator’, which controls many aspects of differentiation (reviewed by [35, 36]), stemness [37], metabolism [38, 39] and apoptosis [40], while the latter, belonging to the forkhead box (FOX) superfamily of transcription factors, is a protagonist of cell cycle progression, regulating genes involved in G1/S as well as G2/M transition and correct mitosis execution [41]. It is not surprising that the deregulation of these two transcription factors culminates in tumour initiation and progression, and that the upregulation mediated by Aurora-A may thus lead to altered signalling cascades in several types of neoplasm.

**Fig. 2:**
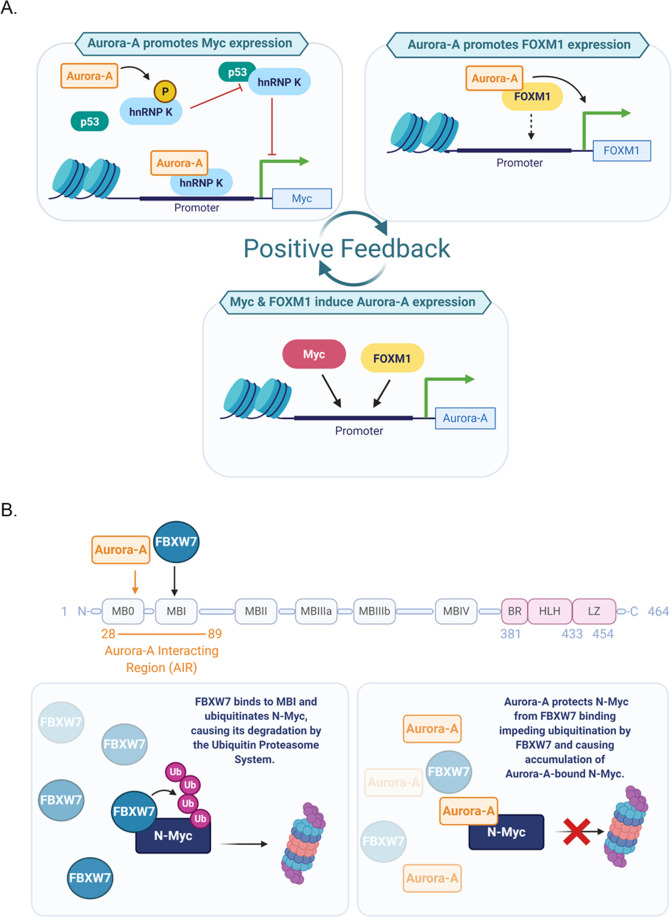
Aurora-A functions in regulating transcription and stability of oncoproteins. **A** Aurora-A can increase Myc expression directly, through promoter shift, in combination with hnRNP K; a potential indirect effect, also mediated by hnRNP K, may also represent an indirect Aurora-A contribution to elevated Myc levels. The right panel schematises the transcriptional effect of Aurora-A on *FOXM1* gene expression. The dashed arrow indicates potential direct binding of an Aurora-A/FOXM1 complex to the *FOXM1* promoter. In turn, Myc and *FOXM1* can upregulate *AURKA* gene expression. **B** The N-Myc protein sequence is schematically represented on top; myc boxes and DNA-binding domains are indicated, as well as the binding regions to Aurora-A and FBXW7. FBXW7 recognises and ubiquitinates N-Myc, inducing proteasome-mediated degradation (lower left panel). Aurora-A binding protects N-Myc from FBXW7-mediated degradation (lower right panel). Created with BioRender.com.

Interestingly, Aurora-A overexpression yields increased levels of Myc and FOXM1 independently of its kinase activity. Indeed, the wild-type kinase and inactive mutants (Kinase Dead; D274N) equally induced the expression of both oncogenes, while their levels decreased following Aurora-A depletion, but not VX680- or MLN8237-mediated inhibition [28, 33]. Intriguingly, despite the same consequences on their expression, Aurora-A regulates the transcription of Myc and FOXM1 through distinct mechanisms (Fig. 2A).

*MYCC* gene is transcribed from two different promoters, P1 and P2, with P2 being preferentially used under physiological conditions, while in some cases, such as in Burkitt’s lymphoma, a shift in P1/P2 promoter usage occurs [42–44] resulting in altered regulation of Myc expression and its increased levels. The nuclear fraction of Aurora-A, but not the cytoplasmic one, induces a P1/P2 shift in promoter usage, further supporting the notion that the nuclear pool of the kinase has transcription activity and suggesting that this *MYCC* regulation also occurs in BCSC [28]. Aurora-A lacks DNA-binding sites, and requires partners to exert its transactivation transcription functions (Fig. 2A). The ribonucleoprotein hnRNP K was identified as the key mediator on the *MYCC* promoter, where an Aurora-A/hnRNP K complex was revealed by re-ChIP assays. Depletion of hnRNP K impairs Aurora-A recruitment on the *MYCC* promoter, but not vice versa. Consistently, nuclear localisation of overexpressed Aurora-A does not lead to an increase in the CD24^low^/CD44^high^ cell population, nor to *MYCC* promoter usage shift towards P1, upon hnRNP K depletion. Thus, the kinase requires hnRNP K to target the *MYCC* promoter and induce the BCSC phenotype [28].

Interestingly, a link between Aurora-A and hnRNP K was previously shown [45], with the ribonucleoprotein being phosphorylated by the kinase on serine 379, an event that disrupts its interaction with p53, of which hnRNP K is also a transcriptional co-activator [45], while not influencing *MYCC* promoter activation [28]. Since p53 negatively controls the expression of multiple cell cycle genes, including *MYCC* [46, 47], this may represent an additional route through which Aurora-A and hnRNP K regulate *MYCC* expression (Fig. 2A, upper left box).

A similar transcription regulatory mechanism involves Aurora-A and the FOXM1 oncoprotein, in BCSC proliferation (Fig. 2A, upper right box). The region +1/+300 of the FOXM1 promoter is required for Aurora-A-mediated transactivation [33]; interestingly, this region contains a forkhead responsive element, suggesting a co-operation of Aurora-A and FOXM1 itself on the *FOXM1* promoter. Supporting this hypothesis, reporter luciferase assays showed that only in presence of FOXM1 can the kinase regulate *FOXM1* promoter transcription [33], similarly to what was observed for Myc expression upon hnRNP K depletion [28]. In turn, both FOXM1 and Myc are able to bind the *AURKA* promoter and activate its transcription, suggesting the existence of a positive feedback loop (Fig. 2A), in which Aurora-A promotes FOXM1 and Myc expression and vice versa [33, 48, 49]. Accordingly, their expression patterns oscillate upon their respective depletion or overexpression; interestingly, depletion of FOXM1 in MDA-MB-231 (high Myc) breast cancer cells was able to reduce not only Aurora-A levels, consistent with the FOXM1 transcription regulation of *AURKA* gene, but also Myc levels [33, 48]. These observations strongly suggest the intriguing hypothesis that both Myc and FOXM1 pathways display an interdependency on Aurora-A, and converge in a synergistic action on the CD24^low^/CD44^high^ BCSC phenotype.

Although further studies are needed to clarify the pathways activated downstream of interphase excess of Aurora-A, evidence summarised so far clearly indicates that its nuclear localisation correlates with oncogenic properties of BCSC, at least partially due to positive regulation of Myc [28] and FOXM1 [33] levels.

## Aurora-A acts to promote stability of oncoproteins

In addition to its involvement in oncogene transcription and expression, Aurora-A was also shown to have a kinase-independent role in the stabilisation of oncoproteins required for cancer cell proliferation.

As for TNBC, in which Aurora-A plays a pivotal role in maintenance of cancer cell proliferation in concert with FOXM1, the kinase is important for the tumour growth of high-risk *MYCN*-amplified neuroblastoma, a subtype of neuroblastoma in which the *MYCN* gene amplification is predictive of higher aggressiveness and poor outcome [50–52]. In fact, shRNA-mediated depletion of Aurora-A led to reduced cellular proliferation and colony formation in both N-Myc- and FOXM1-addicted cancer cells [53, 54]. The reduction in FOXM1 and N-Myc levels is the critical mechanism by which depletion of Aurora-A inhibits tumour proliferation, as confirmation of its interdependence with the two oncoproteins.

Intriguingly, for both neuroblastoma and TNBC, Aurora-A appears to regulate the ubiquitination of the oncoprotein in order to fulfil its pro-tumorigenic function in cancer cells. High levels of Aurora-A increase the half-life of the N-Myc protein by protecting it from proteasome-dependent degradation [54–56], while a reduction in FOXM1 protein half-life was reported following Aurora-A RNAi [53]. Therefore, Aurora-A overexpression augments the oncogenic expression profile of these oncoproteins by mediating their stabilisation. Below, we summarise what has been clarified about the mechanisms through which Aurora-A manages to maintain high levels of N-Myc and FOXM1.

A series of publications has described an important effect of Aurora-A on N-Myc stability [54–57], and evidence exists that this is true for Myc too [58]. In neuronal progenitor cells, N-Myc is phosphorylated at T58 by GSK3β after a priming phosphorylation at S62 by the mitotic cyclin B/CDK1 complex [59]. N-Myc phosphorylated at both sites is recognised by the E3 ubiquitin ligase FBXW7 and marked for proteasomal degradation, with the T58A mutation being sufficient to interfere with this process [60]. In *MYCN*-amplified neuroblastoma, binding of Aurora-A to an overlapping region reduces FBXW7 binding to N-Myc. This results in N-Myc protein protection and stabilisation [54, 56] (Fig. 2B). The capability of Aurora-A to stabilise N-Myc is independent of its kinase activity, since eight different mutant alleles of Aurora-A, all of which have been previously reported to be deficient in kinase activity, were able, as the wild-type kinase, to stabilise N-Myc [54].

Despite its stabilisation of N-Myc, Aurora-A appears to promote the accumulation of ubiquitin conjugates on N-Myc [54] (Fig. 2B). These may be so-called ‘unconventional chains’ that are degraded less efficiently by the proteasome with respect to K48-linked or branched poly-ubiquitin chains [54, 61], or that may have functional significance other than proteasome-mediated degradation and specify an alternative fate for the N-Myc protein [62]. It is therefore possible that Aurora-A interaction modifies the balance of ubiquitin linkages on N-Myc by influencing access of the ubiquitination machinery. We note that UBE2C, an E2 enzyme whose coding gene is located, as is the *AURKA* genomic locus, in the 20q chromosome arm frequently amplified in tumours, may represent an interesting candidate; indeed increased Aurora-A and UBE2C levels in cancer positively correlate, with the two genes occurring within cancer-related signatures, and an interaction between them has been reported [63–65]. Moreover, UBE2C is among the top ten genes with similar expression pattern to Aurora-A in tumour cells, according to GEPIA meta-analysis (http://gepia.cancer-pku.cn).

Yang et al. [53] proposed that Aurora-A binding to FOXM1 reduces its ubiquitin-dependent turnover in breast cancer cell lines. Similarly to what is described for N-Myc in neuroblastoma, this Aurora-A function would be independent of its kinase activity in TNBC, since the administration of the Aurora kinase inhibitor VX680 in MDA-MB-231 cells does not cause a reduction in FOXM1 protein levels. A number of E3 ubiquitin ligases contributing to FOXM1 regulation have been identified (reviewed by [66]) and it is not known yet which of these ubiquitination pathways would be regulated by Aurora-A, and if the mechanism would resemble that described for the better known stabilisation effect on MYC family proteins.

Interestingly, the isoforms of FBXW7 used for experimental demonstration of the competition between ubiquitin ligase and Aurora-A for the binding to N-Myc (FBXW7α and FBXW7γ) are localised in the nucleus [67], arguing that the nuclear fraction of the kinase may be responsible for N-Myc protein stabilisation. We also found evidence that RNF168, an E3 ubiquitin ligase able to ubiquitinate FOXM1 in breast cancer, displays nuclear localisation [68]. Despite no evidence yet existing to show that Aurora-A can compete with FOXM1 ubiquitin ligases, it will be interesting to investigate the potential interplay between nuclear Aurora-A and RNF168 in regulating FOXM1 levels in breast cancer.

## Can Aurora-A mutations in cancer impact on its localisation?

Despite several studies evaluating the oncogenic potential of the nuclear localisation of Aurora-A, the import/export trafficking of the kinase through the nucleus is poorly explored and underlying mechanisms have not been clarified. Nuclear accumulation of overexpressed Aurora-A-GFP was observed to different extents depending on the cellular system (HeLa versus Xenopus XL2 cells) and was modulated by Leptomycin B administration, an inhibitor of CRM1-mediated nuclear export [69]. Deletion analyses indicated that the 333-383 region of the kinase is required for nuclear accumulation of GFP-tagged Aurora-A. In this work, the authors suggest that the portion required for nuclear export lies within aa 1-333 [28], consistent with an earlier study indicating that determinants of Aurora-A cytoplasmic localisation lie in the N-terminal disordered region of the kinase [69]. Interestingly, the first 30 aa of Aurora-A are important for its mitochondrial localisation, and have been proposed to contain an atypical mitochondrial targeting sequence, which for its full targeting function requires proteolytic cleavage, post-translational modifications and/or specific interactions [7, 8].

**Fig. 3:**
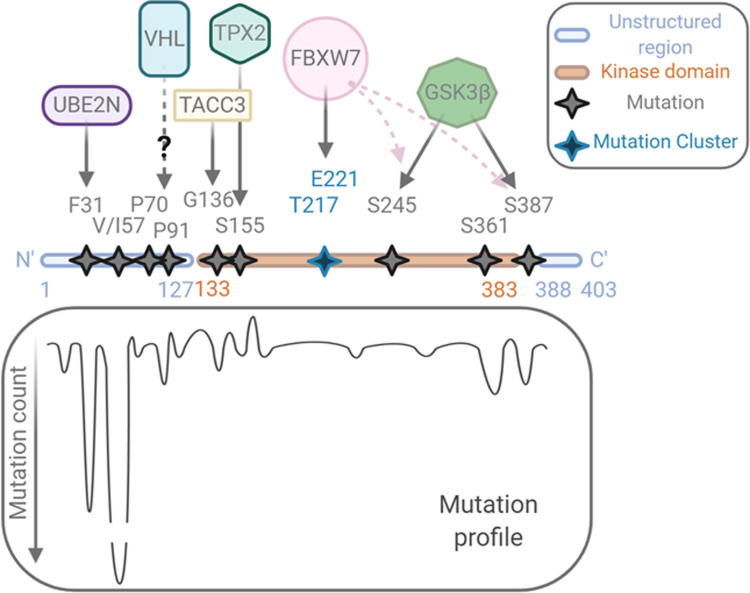
Overview of cancer-associated Aurora-A mutations and their functional relevance. The mutation profile of Aurora-A in cancer is shown, together with the potential partners whose physical or functional interaction can be influenced by the mutations. Data from COSMIC v92 release (August 2020) lists a total of 455 unique samples with mutations. Created with BioRender.com.

Due to the importance of nuclear Aurora-A localisation in mediating non-mitotic oncogenic functions, we looked for evidence that link somatic mutations in the kinase, as reported in the Catalogue Of Somatic Mutations In Cancer [70, 71] (COSMIC v.92, https://cancer.sanger.ac.uk/cosmic), and their reported localisation in the literature. Overall, this search highlighted a cluster of mutations in the disordered N-terminal and C-terminal regions (Fig. 3), but with little or no indication of how these mutations affect Aurora-A localisation. We found instead that several of the mutations could be linked to Aurora-A interaction with binding partners that could regulate either localisation or stability of the protein (Fig. 3). Stability regulators might have an indirect effect on localisation by promoting accumulation or degradation in some subcellular compartments. In addition, enhanced nuclear localisation of Aurora-A could result from altered activity of the nuclear import and export machinery in specific cancer types. However, we did not find evidence directly linking alterations in CRM1, the only transport machinery factor so-far investigated for Aurora-A localisation, with nuclear localisation of Aurora-A in cancer databases.

We looked at mutations of residues that are known to affect the ability of Aurora-A to interact with established binding partners (Fig. 3). A well-characterised cancer-associated mutation occurs at S155R (count of three, as of v92 release, 2020), which shows decreased activity in vitro as a result of loss of interaction with TPX2 and therefore decreased Aurora-A at the mitotic spindle [72]. Our research of the COSMIC database also highlighted S361* truncation (count of five), which results in an inactive and possibly unstable protein [72]. Furthermore, we found the residue G136, which is involved in the interaction with TACC3 during mitosis [73] to be mutated to A (count of four). No information regarding the subcellular localisation of this mutant could be found. Although these mutations lead to decreased mitotic kinase activity of Aurora-A, which depends on TPX2 and TACC3, their contribution to interphase activity has not been assessed. We observe that interaction with TPX2 (nuclear) and TACC3 (cytosolic) could be parameters influencing the localisation of Aurora-A in interphase, when kinase-independent roles may be critical. Lastly, Aurora-A, like N-Myc, is a known substrate for the FBXW7 ubiquitin ligase, shown to interact with FBXW7 through residues T217 and E221 [74]. COSMIC reveals small clusters of low count mutations in neighbouring amino acids. S387 and S245 are phosphorylation sites for GSK3β and are required for FBXW7 phospho-degron recognition and ubiquitination; these are indeed found mutated: S245P (one count) and S387L (two counts), as well as additional surrounding residues (Y246H, three counts; S249L, four counts; A385P/T, six counts; S388*/L, three counts). The well-characterised interaction between N-Myc and Aurora-A [54], discussed in a previous section, has always been described as a stabilising role of Aurora-A for N-Myc. Since both proteins are targets of the same F-box protein, it is possible that they stabilise each other by their interaction, with mutual protection from FBXW7-mediated degradation augmenting their oncogenic capability.

Two known allelic variants of Aurora-A that give rise to F31I and I57V substitutions in its N-terminal disordered region are also classed as somatic mutations associated with cancer by the COSMIC database. Multiple studies testing the significance of these alleles in cancer risk have led to conflicting results, resolved by a meta-analysis which concluded that I31 and V57 can each confer some ethnicity-dependent risk [75]. The functional significance of I57V is not known. Interestingly, the study which first associated the Ile31 version of Aurora-A with increased aneuploidy and stronger transforming properties in colon cancers [76] showed the mutant protein to have reduced interaction with ubiquitin-conjugating enzyme UBE2N and strongly reduced ubiquitination compared to the wild-type Phe31 version [76]. Since UBE2N is reported to be nuclear (http://www.proteinatlas.org/, [77]), mutation to isoleucine at position 31 might stabilise Aurora-A in a nuclear setting to promote its transforming abilities. Finally, two N-terminal prolines of Aurora-A also feature in the COSMIC list: P70L and P91L/Q (counts of two and five, respectively). Whilst these mutants have not yet been characterised, we note the recently published interaction between Aurora-A and the E3 ligase von Hippel–Lindau (VHL, [78]) that recognises its substrates through hydroxylated prolines [79]. Notably, the cancer-associated VHL mutant is unable to degrade wild-type Aurora-A [78].

Together these investigations suggest that cancer-associated Aurora-A mutations may affect the stability of the kinase, or interaction with specific activators, that can, in turn, be reflected in abnormal accumulation in distinct cellular compartments.

## Targeting Aurora-A kinase-independent functions: a new therapeutic challenge

Given the potential of Aurora-A as a target for cancer therapy, several inhibitors of its catalytic activity have been developed over the years, in order to impair its mitotic function and hence cell division [19, 22]. Indeed, all the Aurora-A inhibitors that have entered clinical trials act as ATP-competitors in the active site pocket, which is highly conserved among human kinases. Despite great efficacy (MLN8237 [80]), increased selectivity (LY3295668 [81]) and elevated potency (MK-5108 has an IC_50_ of 0.064 nM [82]), the ATP-competitive inhibitors are well known for exhibiting high promiscuity [83–85]. To date, they have not met expectations raised in cellular studies.

**Fig. 4:**
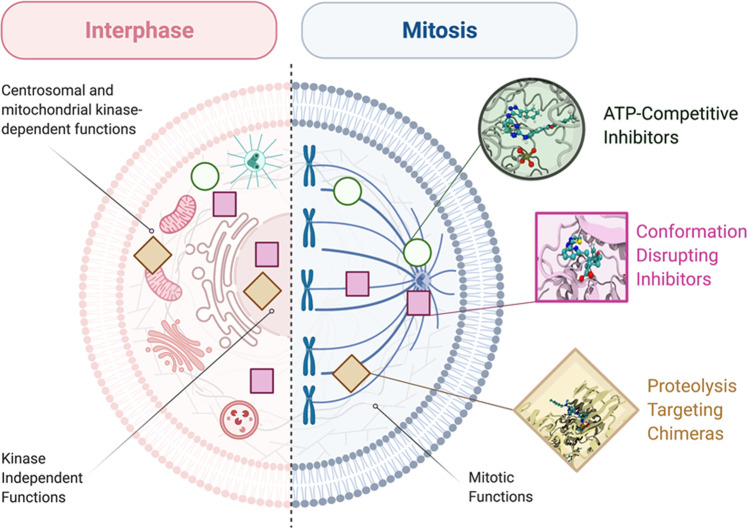
Targeting of interphase and mitotic functions of Aurora-A. Aurora-A functions in an interphase (left) or mitotic (right) cell are schematised, with indication of their dependence on specifically localised Aurora-A pools or on kinase activity. On this basis, we propose that different classes of Aurora-A inhibitors (schematised on the right and indicated by symbols within the cell) can differently affect different Aurora-A functions. Created with BioRender.com.

How can the poor efficacy in clinical trials of classical Aurora-A kinase inhibitors be explained? This gap has been generally attributed to a relatively slow cell proliferation rate in solid tumours and low selectivity [22, 86]. However, the accumulating evidence for kinase-independent oncogenic roles of nuclear Aurora-A suggest an alternative explanation: that poor response to inhibitors instead reflects a failure to target these kinase-independent roles. In this view, ATP-competitive molecules may leave a relevant Aurora-A population untargeted. Targeting not only kinase-dependent Aurora-A functions, but also the emerging kinase-independent ones, in order to suppress the oncogenic ability of Aurora-A, appears as a new challenge in cancer treatment. Possible innovative solutions may come from combined therapies, disruption of Aurora-A interactions with specific partners—including those influencing localisation to specific subcellular sites—and total depletion of the kinase, as we detail in the following paragraphs (schematised in Fig. 4). Parallel studies aimed at characterising Aurora-A status in clinical samples may help driving patient stratification for a more effective response to such treatments.

The combination of various treatments has been demonstrated to be effective in breast cancer, since co-inhibition of Aurora-A and FOXM1 effectively breaks the feedback loop and interferes with the kinase and non-kinase functions of Aurora-A in TNBC, targeting, respectively, both cytoplasmic (kinase dependent) and nuclear (kinase independent) functions [33]. Results showed that combinatorial treatment with Aurora-A kinase inhibitor AKI6038 and FOXM1 inhibitor thiostrepton in MDA-MB-231 cells yielded strong growth inhibition and suppression of colony, mammosphere and xenograft tumour formation, with respect to the single drugs administered alone, confirming that inhibition of Aurora-A and FOXM1 synergistically impairs the growth of BCSC [33].

Specific Aurora-A functions not relying on kinase activity may also be impaired through inhibition of relevant protein–protein interactions. A well-studied example is the Aurora-A/N-Myc interaction in neuroblastoma. Indeed, N-Myc-addicted neuroblastoma cells are sensitive to a specific sub-class of ATP-competitive Aurora-A kinase inhibitors able to induce a conformational change of the kinase, resulting in disruption of the Aurora-A/N-Myc complex [55–57]. The interaction between Aurora-A and N-Myc requires an open conformation of the activation loop of the kinase; conversely, these so-called conformation disrupting inhibitors (CD-inhibitors), such as CD532 and Alisertib (MLN8237), are able to block the activation loop in a closed conformation [87], interfering with Aurora-A kinase activity and, at the same time, with the Aurora-A/N-Myc interaction. Although this has so far been shown only at high concentrations of the drugs (low micromolar range) with limited usefulness in vivo, it opens up the interesting possibility of identifying Aurora-A kinase inhibitors able to effectively block both kinase-dependent and N-Myc-related kinase-independent functions [55–57].

The disruption of the Aurora-A/N-Myc complex allowed by CD-inhibitors can also promote association of N-Myc with other partners (RAD21, TOP2A, TFIIIC5) identified as N-Myc interactors throughout the G1 phase of the cell cycle [88]. Indeed, the Aurora-A/N-Myc interaction may act as a protective mechanism during DNA replication to prevent unscheduled transcriptional activity of N-Myc. Consistently, CD-inhibitors induced increased levels of the marker of recovery of collapsed replication forks phospho-RPA32(S33), suggesting an S phase progression perturbation [88]. Furthermore, it has been recently demonstrated that N-Myc-dependent activity of Aurora-A is required for the phosphorylation of histone H3 at S10 in S phase, to antagonise N-Myc-dependent transcription–replication conflicts. Co-inhibition of Aurora-A and ATR (required for the stability of replication stalled forks) causes tumour regression and immune system activation in *MYCN*-amplified neuroblastoma animal models [89]. In addition, N-Myc-independent functions of Aurora-A on stalled forks (kinase dependent [90]) and in the activation of replication origins (kinase independent [91]) were recently discovered. Interestingly, co-treatment with replication initiation inhibitors and CD532, but not with compounds targeting only the kinase activity of Aurora-A, synergistically acted to arrest cell proliferation [91].

In the last few years, specific inhibitors of Aurora-A binding to its major activator TPX2 have also been developed, with first cellular studies indicating that they are able to inhibit Aurora-A autophosphorylation and interfere with proper organisation of the mitotic spindle [92–94]. New efforts directed towards disrupting interactions relevant to interphase functions, and specifically nuclear functions of Aurora-A, may constitute an interesting future perspective.

Another therapeutic approach that has recently gained traction is the possibility of using small molecule drugs for elimination of a target protein through ‘hijacking’ a cellular E3 ligase to ubiquitinate it, leading to its ubiquitin-mediated destruction by the 26S proteasome [95]. One of the clear advantages of this strategy is to suppress all functions of a target protein, and in the case of Aurora-A, both kinase-dependent and -independent functions. Furthermore, the catalytic nature of such small molecules (Proteolysis Targeting Chimera, ‘PROTACs’), which act to mediate ubiquitination reactions through transient ternary complex formation between the participants [96] rather than through occupancy of a binding pocket, raises the prospect that they can act at low doses and sub-optimal affinities for the target. Two recent studies have tested PROTACs carrying the MLN8237 ‘warhead’ linked to ligands for the E3 Cereblon complex [97, 98]. These tools are found to efficiently eliminate Aurora-A protein from the cell, resulting in phenotypes distinct from those produced by MLN8237 treatment. Thus, Adhikari et al. [97] show that MLN8237-treated cells arrested in mitosis, whilst PROTAC-treated cells avoid mitotic arrest, arresting in S phase of the cell cycle instead. Our own study shows that mitotic spindle assembly can take place in the presence of an Aurora-A PROTAC due to differential targeting of subcellular pools of the target in mitosis that leaves a centrosomal pool intact [3, 98]. Both studies indicate that targeting its kinase-independent roles provides a novel therapeutic context for drugging Aurora-A. Furthermore, a recent chemo-proteomic study of more than 200 protein kinases identified Aurora-A as amongst those showing highest susceptibility to targeted degradation [99], indicating the potential nuclear oncogenic pool of Aurora-A as a promising therapeutic target for the future design of targeted protein degradation tools.

Although development of Aurora-A ATP-competitive kinase inhibitors has so far dominated the approaches to target mitotic Aurora-A in cancer, it is now clear that oncogenic pools of the kinase which act in interphase and in specific compartments—as for the nuclear functions described in this review—may not be effectively targeted by such strategies. Developing new strategies able to specifically reach these pools will contribute to better exploiting Aurora-A as a target for anti-cancer therapies.
